# Vaccine-induced immune thrombotic thrombocytopenia: what do we know hitherto?

**DOI:** 10.3389/fmed.2023.1155727

**Published:** 2023-05-16

**Authors:** Renat Roytenberg, Adolfo García-Sastre, Wei Li

**Affiliations:** ^1^Department of Biomedical Sciences, Joan C. Edwards School of Medicine at Marshall University, Huntington, WV, United States; ^2^Department of Microbiology, Icahn School of Medicine at Mount Sinai, New York, NY, United States; ^3^Department of Medicine, Division of Infectious Diseases, Icahn School of Medicine at Mount Sinai, New York, NY, United States; ^4^The Tisch Cancer Institute, Icahn School of Medicine at Mount Sinai, New York, NY, United States; ^5^Department of Pathology, Molecular and Cell-Based Medicine, Icahn School of Medicine at Mount Sinai, New York, NY, United States; ^6^Global Health and Emerging Pathogens Institute, Icahn School of Medicine at Mount Sinai, New York, NY, United States

**Keywords:** VITT, COVID-19 vaccine, platelet factor 4, spike protein, PF4/polyanion immune complex

## Abstract

Vaccine-induced immune thrombotic thrombocytopenia (VITT), also known as thrombosis with thrombocytopenia syndrome, is a catastrophic and life-threatening reaction to coronavirus disease 2019 (COVID-19) vaccines, which occurs disproportionately in response to vaccination with non-replicating adenovirus vector (AV) vaccines. The mechanism of VITT is not well defined and it has not been resolved why cases of VITT are predominated by vaccination with AV vaccines. However, virtually all VITT patients have positive platelet-activating anti-platelet factor 4 (PF4) antibody titers. Subsequently, platelets are activated and depleted in an Fcγ-receptor IIa (FcγRIIa or CD32a)-dependent manner, but it is not clear why or how the anti-PF4 response is mounted. This review describes the pathogenesis of VITT and provides insight into possible mechanisms that prompt the formation of a PF4/polyanion complex, which drives VITT pathology, as an amalgam of current experimental data or hypotheses.

## 1. Introduction

Severe acute respiratory syndrome coronavirus 2 (SARS-CoV-2) is the causative agent of coronavirus disease 2019 (COVID-19) (1). SARS-CoV-2 uses its spike protein (SP), an envelope glycoprotein, to enter human cells via angiotensin converting enzyme 2 (ACE2) (2). Ninety percent of neutralizing antibodies in COVID-19 patient sera are directed toward the receptor binding domain (RBD) of SP (3), and thus vaccine manufacturers have developed vaccines targeting the SP. There are eight vaccines directed against the SARS-CoV-2 SP which cover the majority of COVID-19 vaccines distributed and administered globally (4) (Table 1). These vaccines utilize different methods to mount immune responses, including mRNA, adenovirus vector (AV), inactivated virus, or recombinant adjuvanted protein. The eligible COVID-19 vaccines used in the Western world, including ChAdOx1 nCoV-19 by Oxford/AstraZeneca, Ad26.COV2.S by Johnson & Johnson/Janssen (J&J), BNT162b2 by BioNTech-Pfizer, and mRNA-1273 by Moderna, use the same variant of native, full length SP as an immunogen, Wuhan-Hu-1 (16).

**Table 1:** Characteristics of worldwide COVID-19 vaccines and their relation to VITT.

Manufacturer	Vaccine name	Vaccine type	Vaccine efficacy	VITT Incidence, as of Q1 2022	VITT mortality rate, as of Q1 2022	Total # of vaccines administered in the USA or EU (in millions) (5)
Moderna	mRNA-1273	mRNA (Lipid nanoparticle)	94% (6)	Low	n/a	398
BioNTech-Pfizer	BNT162b2	mRNA (Lipid nanoparticle)	95% (6)	Low	n/a	1,044
Oxford/AstraZeneca	ChAdOx1 nCoV-19	Adenoviral vector (Y25)	63% (6)	1/64,000 (7), 1/125,000 (8)	18% (7)	67
Johnson & Johnson/Janssen	Ad26.COV2.S	Adenoviral vector (Ad26)	67% (9)	1/310,000 (7), 1/200,000 (8)	15% (7, 10)	37
Gamaleya Research Institute of Epidemiology and Microbiology	Sputnik V	Adenoviral vector (Ad26 and Ad5)	91% (6)	Low (11)	n/a	1.8
CanSino Biologics	Ad5-nCoV-S	Adenoviral vector (Ad5)	58% (12)	n/a	n/a	n/a
Sinovac Biotech	CoronaVac	Inactivated virus	50% (6)	n/a	n/a	0.007
Sinopharm	BBIBP-CorV	Inactivated virus	78% (6)	Low (13)	n/a	2.3
Bharat Biotech	BBV152	Inactivated virus	78% (14)	n/a	n/a	0.0001
NovaVax	NVX-CoV2373	Adjuvanted protein	90% (15)	n/a	n/a	0.29

To date, mRNA and AV vaccines have been approved for use by regulatory groups across the world, including in multiple Western nations, and have been highly efficacious in reducing COVID-19-associated thrombotic events such as myocardial infarction (17), and COVID-19-related hospitalization and mortality (18–20). Despite the fast tracking of clinical trials, eligible COVID-19 vaccines have an acceptable short-term safety profile as of the first quarter of 2022 (21). However, a meta-analysis of 58 COVID-19 vaccine efficacy studies showed that severe adverse events occurred at 4 per 100,000 vaccinations (22). In late February 2021, reports of thrombosis with thrombocytopenia occurring after vaccination with ChAdOx1 nCoV-19 led to the temporary suspension of the use of ChAdOx1 nCoV-19 in multiple European countries (23). By March 10, 2021, 30 cases of thromboembolic events in response to five million ChAdOx1 nCoV-19 vaccinations were reported by the European Medicines Agency (24). Subsequently, a new concept termed vaccine-induced immune thrombotic thrombocytopenia (VITT) or thrombosis with thrombocytopenia syndrome (TTS) was first coined by two European groups in April 2021 (25, 26). The U.S. Food and Drug Administration (FDA) and Centers for Disease Control and Prevention (CDC) suggested pausing the administration of Ad26.COV2.S in April 2021 due to six reports of TTS after 6.8 million doses were administered. Soon afterwards, in December 2021, the CDC recommended against the use of Ad26.COV2.S. In May 2022, the FDA officially limited the authorized use of Ad26.COV2.S to adults for whom other authorized COVID-19 vaccines were not accessible or clinically appropriate, or for adults who declined other authorized COVID-19 vaccine options (7). In comparison to the use of AV vaccines, particularly ChAdOx1 nCoV-19 and Ad26.COV2.S, the use of the widely-approved mRNA-based vaccines, BNT162b2 and mRNA-1273, has not led to regulatory warnings or limitations concerning VITT, although there are scarce case reports that document mRNA-based COVID-19 vaccines causing VITT (27–30). Thus far, the mechanism of VITT is not yet clearly defined. In this review, we will provide insight into possible mechanisms that drive VITT. We will also describe its epidemiology and pathology, compare its diagnostic tests, and discuss its therapeutic paradigm.

## 2. VITT characterization

VITT is a potentially fatal consequence of COVID-19 vaccination in which blood clotting and thrombocytopenia are induced via an immune response to the endogenous protein, platelet factor 4 (PF4) (31). The vast majority of dually thrombotic and thrombocytopenic responses to COVID-19 vaccination are VITT (31). Per the American Society of Hematology (32), which corresponds its classification of VITT with the Brighton Collaboration Criteria (33) and the UK Hematology Expert Group (34), VITT is classified by five criteria: (1) COVID-19 vaccine administered 4 to 42 days prior to symptom onset; (2) venous or arterial thrombosis (usually cerebral or abdominal); (3) thrombocytopenia (platelet count <1.50E+12/L); (4) dramatically elevated D-dimer (> 4 times the upper limit of normal); and (5) positive anti-PF4 antibody titers. VITT-associated thromboses happen at unusual sites including the cerebral venous sinus and splanchnic veins (24, 25, 35, 36). In fact, VITT is largely characterized by cerebral venous sinus thrombosis (CVST) with thrombocytopenia (35, 37). VITT patients commonly present with severe headache (37, 38). However, Salih et al. suggest that severe headache may not be a symptom secondary to CVST/VITT, as their data show the onset of severe headache occurring before any cerebrovascular complications in suspected VITT patients. They consequently coined the term, “pre-VITT syndrome,” indicating the critical period after vaccination with AV COVID-19 vaccines but preceding thrombotic complications where patients may present with severe headache, elevated D-dimer, and/or positive anti-PF4 IgG titer (38, 39).

## 3. Incidence and mortality in VITT

The incidence for VITT varies by the reporting country and the COVID-19 vaccine administered (Table 1). According to the European Medicines Agency, 98.5% of VITT cases are caused by AV vaccination, with ChAdOx1 nCoV-19 and Ad26.COV2.S contributing to 90 and 8.5% of cases, respectively (35). VITT occurs in 1/64,000 (7) to 1/125,000 (8) inoculated with ChAdOx1 nCoV-19 and in 1/200,000 (8) to 1/310,000 (7) inoculated with Ad26.COV2.S. Regarding vaccination with ChAdOx1 nCoV-19, VITT can occur in response to the first or second dose, with the vast majority of cases originating from the first dose (7). The incidence of VITT is extremely low following the use of mRNA-vaccines (10, 40), including BNT162b2 and mRNA-1273. There have been two reports of VITT from the Argentinian Ministry of Health following inoculation with Sputnik V (11), which utilizes human adenovirus 26 (Ad26) in its first administration and human adenovirus 5 (Ad5) in its second administration. There are no confirmed reports on VITT that are associated with Ad5-nCoV-S, the adenoviral-vectored vaccine manufactured by CanSino Biologics or inactivated whole-virion COVID-19 vaccines manufactured by Sinovac Biotech (CoronaVac) and Bharat Biotech (BBV152) as of the third quarter of 2022. However, two VITT cases have been reported in patients vaccinated with BBIBP-CorV by Sinopharm (13), an inactivated whole-virion COVID-19 vaccine, with hundreds of millions of BBIBP-CorV distributed to multiple countries. Therefore, in this review, we will only focus on the mRNA and AV vaccines used in the Western world (as well as globally), particularly the AV vaccines.

The mortality rate of VITT was as high as 47% before March 2021 (35), and was reported to be 40% in the acute phase based on a study that recruited 107 CVST-VITT patients by February 10, 2022 (36). Early recognition of VITT or “pre-VITT syndrome” is critical for a favorable prognosis. Importantly, the mortality rate has more than halved to 22% after March 2021 due to early identification (25, 35). In fact, Australia has reported a mortality rate as low as 5% for VITT, which is likely attributed to an educational program on VITT accompanying their COVID-19 vaccination efforts (41). The meta-analysis by Kim et al., which evaluated 18 VITT studies conducted in 2021, found an average mortality rate of 32% for VITT (37). They also showed that CVST, deep vein thrombosis/pulmonary thromboembolism, and splanchnic vein thrombosis occurred in 54, 36, and 19% of VITT patients, respectively; and the incidence of CVST is over 25 times higher following ChAdOx1 nCoV-19 vaccination compared to the pre-pandemic general population. Intracranial hemorrhage and extracranial thrombosis were found to be associated with CVST in VITT patients, at rates of 47 and 33%, respectively (37). Patients with a high degree of thrombocytopenia have the poorest prognosis (34). There are no apparent risk factors for VITT (25, 34, 42). However, some studies have claimed a female sex bias (37, 43). Hwang et al. have devised a scoring system to predict mortality in VITT patients called FAPIC (fibrinogen, age, platelet count, ICH, and CVT) based on 64 VITT patients (44). Though FAPIC criteria are mostly comprised of pathological hallmarks of VITT, Hwang et al. indicate that those aged under 60 years old are at a higher risk of mortality. In concordance, there may be an age bias for younger people vaccinated with ChAdOx1 nCoV-19 aged 50 years and lower, where rates of VITT were observed to be doubled compared to those aged over 50 years receiving ChAdOx1 nCoV-19 (7, 34).

Due to its high mortality rate, there is an urgency to spread information on VITT and most importantly, to understand the mechanisms behind this disorder, which will help pave the road to develop novel therapeutic strategies and inspire rigorous vaccine design.

## 4. Attributes of COVID-19 vaccines in correlation to VITT

BNT162b2 and mRNA-1273 are mRNA-based and feature SP transcripts housed in lipid nanoparticles (LNPs) which protect from mRNA degradation, facilitate cell entry, and deliver the transcripts unscathed to ribosomes in the cytoplasm (16). LNPs consist of phospholipids, cholesterol, PEGylated lipids, and ionizable lipids, which act in tandem to stabilize the LNP, prolong its circulation, and effectively deliver the housed mRNA to target cells (e.g., myocytes and antigen presenting cells) (45, 46). LNPs were found to induce robust inflammation in mice (45). Liver and splenic injury were also reported in rats and monkeys after intravenous (IV) injection with LNPs (47). However, the effect of LNPs or mRNA-based COVID-19 vaccines on VITT is relatively negligible due to only several cases of mRNA vaccine-induced VITT reported with billions of mRNA-based COVID-19 vaccines distributed worldwide (10, 30, 40).

ChAdOx1 nCoV-19 and Ad26.COV2.S are DNA-vectored, and therefore undergo more processing steps than mRNA vaccines to express SP. Both ChAdOx1 nCoV-19 and Ad26.COV2.S are administered intramuscularly at a standard dose of 5.0E+10 viral particles (9, 48) in 0.5 mL of solution. However, Ad26.COV2.S requires one dose while ChAdOx1 nCoV-19 requires two. ChAdOx1 nCoV-19 uses chimpanzee adenovirus Y25 as a vector, and is propagated in primary human embryonic kidney cells (HEK293), while Ad26.COV2.S uses human adenovirus 26 derived from human embryonic retinal cells (PER.C6) (16). Ad26.COV2.S uses stabilizing mutations to prevent SP from fusing to cellular membranes while ChAdOx1 nCoV-19 does not employ any stabilizing mutations (16). Therefore, the SP formed by Ad26.COV2.S is both membrane-bound and non-fusogenic, while the SP expressed by ChAdOx1 nCoV-19 retains fusogenicity (49) and may be variably soluble as well (see section “SARS-CoV-2 spike protein as a potential polyanion”) (50). ChAdOx1 nCoV-19 contains a leader sequence at the N-terminal consisting of a signal peptide of human tissue plasminogen activator (tPA) (16, 51, 52), which purportedly increases immunogenicity (16). The tPA leader sequence is expressed and acts as a signaling sequence to traffic its attached protein via the cell secretion pathway. In principle, the leader sequence is cleaved and is degraded within the cell, but could remain attached leading to an unknown fate of the tPA-containing SP (53). Nevertheless, the expression of the tPA leader sequence of ChAdOx1 nCoV-19 in the context of VITT is unexplored.

Codon optimization is a technique used to modify amino acid codon composition without altering the primary amino acid sequence in efforts to increase expression of a nucleic acid (54). Oxford/AstraZeneca, J&J, Pfizer-BioNTech, and Moderna all employ manufacturer-specific approaches to codon optimize their vaccines. However, codon optimization of nucleic acids can lead to the production of novel proteins with unintended biological consequences (54). For example, codon optimizing nucleic acid vaccines can change local rates of translation elongation, which can alter protein folding of a target immunogen (55). In fact, differences in cryptic splicing have been observed in DNA-vectored vaccines as a function of codon optimization, specifically for the SP-encoding sequence in ChAdOx1 nCoV-19 and Ad26.COV2.S (50). Whether differentially spliced SP could play a role in VITT is discussed in Section “SARS-CoV-2 spike protein as a potential polyanion.”

Comparative analysis of ChAdOx1 nCoV-19 and Ad26.COV2.S SARS-CoV-2 reveals that ChAdOx1 nCoV-19 contains significantly more impurities, including host-cell and adenoviral proteins (56, 57). In contrast, mRNA vaccines contain no host-cell or adenoviral protein impurities since mRNA is produced via an *in vitro* cell-free transcription reaction, which uses no animal derived materials (58). Whether viral DNA vectors or manufacturing impurities contribute to VITT is discussed in Section “Adenovirus vector as a potential polyanion” and “Contamination with host-cell and/or adenoviral proteins act as a potential polyanion”, respectively.

Additionally, AV vaccines induce different innate immune responses compared to mRNA-based vaccines, with notable differences including increased acute inflammatory cytokine signatures (59, 60), increased platelet activation (59–61), and that ChAdOx1 nCoV-19-induced antibodies promote neutrophil-mediated phagocytosis (62, 63). One study found that while ChAdOx1 nCoV-19 was less immunogenic than BNT162b2, it was more reactogenic and led to acute increases in inflammatory cytokines such as interferon-γ that coincided with increased platelet activation (60). AV COVID-19 vaccines may facilitate the formation of a pro-inflammatory and pro-thrombotic milieu in VITT, which supports the concept of raising plasma PF4 levels over a threshold to mount an anti-PF4 response (see Section “Platelet factor 4”). Nevertheless, it is unknown whether some threshold of inflammation is required to facilitate the anti-PF4 response in VITT.

## 5. VITT is an anti-PF4 mediated disorder

By definition, all VITT patients have platelet-activating anti-PF4 antibody titers (25, 31, 37, 64–67). The pathogenesis of VITT is still under investigation. Though, the pathology of established anti-PF4 disorders has served as a springboard to elucidate how VITT manifests. For example, VITT has often been compared to classical heparin-induced thrombocytopenia (HIT) (25, 34, 68–70), in which an anti-PF4 response is induced after heparin treatment, and to autoimmune HIT (aHIT), in which an anti-PF4 response is heparin-independent (71, 72). Though VITT can be considered a type of aHIT (71), the sites of thrombosis between VITT and aHIT are drastically different, suggesting a differential pathology. Like VITT patients, HIT patients with the highest degree of thrombocytopenia have the worst prognoses (73). However, unlike VITT patients, CVST is rarely observed in HIT patients (74), and a considerable proportion of HIT patients do not develop thromboses (42, 75). This is possibly due to the quality of VITT anti-PF4 antibodies differing from HIT anti-PF4 antibodies. Biolayer interferometry showed that VITT anti-PF4 antibodies bind more intensely to PF4 and PF4/heparin complexes than HIT anti-PF4 antibodies (76). HIT anti-PF4 antibodies mostly bind different critical sites on PF4 compared to VITT anti-PF4 antibodies as well (76). VITT patients present with oligoclonal or monoclonal anti-PF4 populations, while anti-PF4 populations in HIT patients are polyclonal (65). Additionally, the anti-PF4 response in VITT can generate multiple populations of antibodies, some of which do not have pathogenic platelet-activating capability (65, 66, 77, 78). Interestingly, 4.3% of healthy blood donors have positive anti-PF4 antibody titers without previous therapeutic heparin exposure (79), suggesting that non-pathogenic anti-PF4 responses exist in some individuals (data collected prior to the COVID-19 pandemic). Non-platelet activating anti-PF4 antibody titers have correlated with vaccine titers post ChAdOx1 nCoV-19 vaccination as well (66). Overall, these findings indicate that the generation of platelet-activating anti-PF4 antibodies detected in VITT patients may depend on some antigen resource, which is introduced after vaccination, deeming VITT as a novel and distinct anti-PF4 disorder.

### 5.1. Platelet factor 4

PF4 is a chemokine stored in and released by alpha-granules upon platelet activation. PF4 is known to participate in blood coagulation by neutralizing negatively charged glycosaminoglycans (GAGs) on endothelial cell surfaces, thereby allowing platelets, which have negatively charged surfaces, to associate with endothelium more avidly, thus promoting thrombus formation (80, 81). However, the endogenous hemostatic function of PF4 may not be relevant in the pathology of an anti-PF4 disorder.

It is important to note that a threshold concentration of circulating PF4 is required to mount an anti-PF4 response (82). Plasma titers of PF4 are normally between 4 and 24 ng/mL with a median of 7.4 ng/mL, which are distinctly increased in some diseased conditions but are normal in immune thrombocytopenia (83). Therefore, increased PF4 titers on their own are not responsible for platelet destruction (83). Regardless, to the best of our knowledge, plasma PF4 titers have not been studied in VITT nor COVID-19 patients.

### 5.2. PF4/polyanion complex formation

Though the pathological anti-PF4 response depends on a sufficient titer of circulating PF4, it crucially depends on PF4/polyanion complex formation. PF4 contains a cationic equatorial band rich in lysine and arginine that contribute to form a ring of positive charge (75). This way, PF4 has a high affinity for negatively charged polyanions, such as GAGs (e.g., heparan sulfate, dermatan sulfate), DNA, or polymeric heparin. PF4/complex formation is also dependent on molecular size or length. PF4 was found to form complexes with molecules carrying strong negative charges spaced about 0.5 nm apart along the molecular backbone, while reaching a threshold length that spans about 40% of the circumference of a PF4 tetramer (84). In essence, large and negatively charged molecules can bind clusters of tetrameric PF4, which can effectively lead to the merging of adjacent charge clouds of PF4 tetramers, thus causing a release of energy that induces a conformational change in the individual PF4 tetramers, revealing neoantigen(s) or anti-PF4 binding sites (85). Additionally, although PF4 exists in monomeric, dimeric, and tetrameric forms at equilibrium (86), only tetrameric PF4 has been pathologically relevant in anti-PF4 disorders (87).

PF4/heparin complex formation in the context of HIT has been well studied and discussed in many excellent works (75, 84–86, 88–90). Although VITT patients test strongly positive in PF4/polyanion enzyme immunoassays (77, 91), it is unknown what triggers PF4/polyanion complex formation in VITT. We will focus on polyanion candidates present after COVID-19 vaccination, including SARS-CoV-2 SP, adenoviral vector, and contamination of host cell and viral proteins from AV vaccines, and discuss their potential mechanistic roles in VITT.

#### 5.2.1. SARS-CoV-2 spike protein as a potential polyanion

The SARS-CoV-2 SP is a large molecule (180–200 kDa) with a negative charge that predominates its surface, containing 99 positively and 111 negatively charged amino acids (92, 93). One study found that SARS-CoV-2 RBD and PF4 may bind each other as anti-PF4 antibodies weakly recognize recombinant RBD (94). Though, this same study did not find that an anti-SP polyclonal antibody could recognize PF4. Similarly, using VITT patient sera, Greinacher et al. showed that VITT anti-PF4 antibodies induced by ChAdOx1 nCoV-19 vaccination do not considerably cross-react with recombinant SP/RBD (95). However, it was not investigated whether platelet activation differed if SP and PF4 were combined, as opposed to SP or PF4 used alone, which would test whether SP initiated or potentiated PF4-induced platelet activation of VITT serum-treated platelets. We speculate that the neoepitope of the novel neoantigen in VITT containing PF4 and SP and/or other proteins may not be well recognized by antibodies against its individual components, and antibodies targeting the novel neoepitope in VITT may also not recognize individual components if they are separated from each other.

Greinacher et al. further found that 19 of 222 COVID-19 patients developed anti-PF4 antibodies that were unresponsive or weakly responsive to PF4 (95). Another study by Liu et al. found that anti-PF4 titers correlated with COVID-19 disease severity scores as well as thrombocytopenia (96), albeit anti-PF4 antibodies observed in COVID-19 patients did not have the platelet-activating capability of anti-PF4 antibodies found in HIT or VITT. Additionally, SARS-CoV-2 infection in vaccinated patients with a history of VITT does not restimulate anti-PF4 antibodies (97); and second dosing with COVID-19 vaccines such as BNT162b2, mRNA-1273, and ChAdOx1 nCoV-19 in 26 patients with confirmed cases of VITT did not restimulate anti-PF4 antibodies as well (98). These data suggest that VITT-inducing neoepitopes are complex, since relapses are not triggered by subsequent COVID-19 vaccination or the addition of potential partial components of the supposed PF4/polyanion complex.

SARS-CoV-2 SP is a hematologically-active molecule that can bind GAGs, like heparan sulfate (99–101), damage endothelium (102, 103), and activate platelets (104, 105). Although local myocytes are the target cells of antigenic expression in vaccination via intramuscular (IM) injection, IM delivery of COVID-19 vaccines can lead to endothelial transfection and the expression of SP by endothelium (106, 107). A level of 10.4 ng/mL of circulating SP has been reported in a patient inoculated with mRNA-1273 (97). Circulating SP has also been detected in patients with postvaccine myocarditis vaccinated with BNT162b2 or mRNA-1273 compared to asymptomatic vaccinated control subjects (108). Additionally, the SARS-CoV-2 S1 subunit has been detected in the sera of CD-1 mice treated with ChAdOx1 nCoV-19 via IM injection up to 14 days (109). De Michele et al. showed that using an antibody directed against the S1 subunit decreased VITT serum-induced platelet activation of washed platelets from healthy donors (110). They suggest a three-hit hypothesis where SP activates endothelium, thereby recruiting and activating platelets, and platelets reactively release PF4 which complexes with some neoantigen that augments VITT thrombi and thrombocytopenia. Taken together, these studies suggest that a neoantigen containing PF4, SP (or S1/RBD), and/or additional unknown proteins, may initiate VITT.

The question raised here is why does vaccination with COVID-19 AV vaccines lead to almost all cases of VITT? In addition to AV vaccination causing greater levels of inflammation and platelet activation when compared to mRNA-based vaccination (59–63), differential splicing in codon-optimized SP encoded by AVs may also play a role. Several open reading frames overlap the SP sequence (111). This finding introduces further opportunity for the translation of unwanted proteins through vaccination. Unintended SP transcripts have been detected in ChAdOx1 nCoV-19 open reading frames (50, 112). Additionally, a cell-dependent detection of adenoviral transcripts was described in A549 cells transfected with ChAdOx1 nCoV-19 (112). Using a splice reporter vector system to validate in-silico splicing data, Kowarz et al. found that a ChAdOx1 nCoV-19 codon-optimized SP-containing construct exhibited significantly more cryptic splice events than Ad26.COV2.S codon-optimized SP or wild-type SP-containing constructs. They coined the term “Vaccine-induced COVID-19 mimicry syndrome,” wherein aberrant splicing in Oxford/AstraZeneca’s codon-optimized SP sequence can lead to a truncated SP, lacking a transmembrane domain, which can unintentionally interact with endothelium via ACE2, mimicking COVID-19 symptoms (50). Additionally, the tPA leader sequence in ChAdOx1 nCoV-19 was not cloned in this study, to ensure that the open reading frame matched with the other SP constructs in the study. The circulation of soluble, truncated SP may lead to unknown consequences.

**Figure 1:**
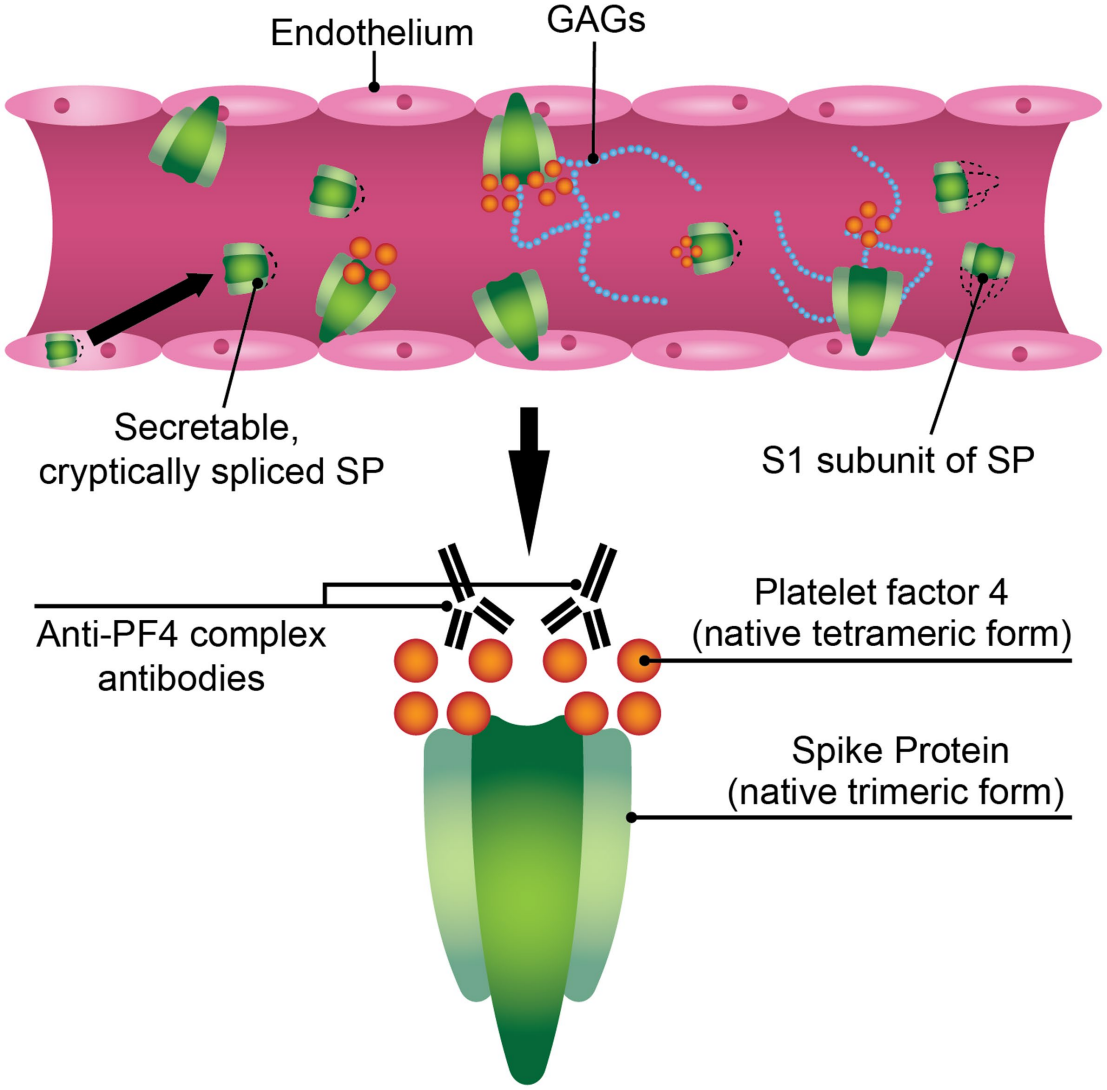
SARS-CoV-2 spike protein (SP) may play a mechanistic role in VITT pathophysiology. The SARS-CoV-2 SP is a hematologically-active molecule that can be expressed in the endothelium post-adenoviral vaccination, where it can interact with components of the bloodstream such as glycosaminoglycans (GAGs) or platelet factor 4 (PF4). A soluble variant of the ChAdOx1 nCoV-19 SP has been reported, where it can directly interact with blood components. The S1 subunit of ChAdOx1 nCoV-19 SP is found in the sera of mice inoculated with ChAdOx1 nCoV-19. These modified or truncated variants of SP may promote PF4/polyanion immune complex formation.

Taken together, SARS-CoV-2 SP is a viable candidate for study in the context of VITT. SP may be facilitating an environment conducive to PF4 release and PF4/polyanion complex formation (Figure 1). SP expressed in endothelium after AV vaccination can activate platelets in the lumen, thereby triggering PF4 release. Since SP can bind GAGs, it is possible that the formation of an electronegatively charged complex protruding from endothelial cells in the blood vessel lumen can bind circulating PF4, causing the formation of neoantigens and subsequently generating an anti-PF4 response. Though the conundrum remains why a minority of those inoculated with COVID-19 AV vaccines experience VITT. VITT may require a set of conditions wherein SP expression in endothelium plays a part but is not sufficient on its own to progress VITT pathology without complementary pathologies (e.g., increased threshold inflammation, higher resting PF4 plasma levels).

#### 5.2.2. Adenovirus vector as a potential polyanion

Adenovirus vector was found to bind platelets directly *in vitro* (113). Recombinant adenoviral vectors have been documented to cause thrombocytopenia *in vivo* as well (114, 115). In fact, a study by one of the largest government-owned transfusion centers in India showed significant deferral rates for plateletpheresis donors who had received one or two doses of ChAdOx1 nCoV-19 due to significantly lower platelet counts compared to controls (116). A study by Nicolai et al. showed that IV, but not IM delivery of ChAdOx1 nCov-19 into mice led to thrombocytopenia (117). Conversely, the study by Stebbings et al. showed mice experiencing transient low platelet counts shortly after ChAdOx1 nCov-19 administration via the IM route (109), albeit these studies used different strains of mice. In comparison, no thrombocytopenia was observed after IV application with another adenovirus strain, ADV-004, which suggests that thrombocytopenia in IV-delivered ChAdOx1 nCov-19 is dependent on direct interaction with adenoviral vector and/or adenovirus subtype (117). Platelet-adenoviral vector aggregates are then recycled in the spleen and platelet remnants are detectable in the marginal zone, where B cells produce circulating antibodies that bind to platelets (117). Additionally, in a study of 631 healthcare workers inoculated with ChAdOx1 nCoV-19, von Willebrand factor (vWF), a marker of endothelial activation and factor of primary hemostasis, was increased by 39.5% (118). Adenovirus can promote platelet clearance in a vWF/P-selectin dependent manner (113), which may potentially contribute to thrombocytopenia in VITT. The role of vWF in VITT is discussed in depth in Section “Other potential mechanisms mediating platelet activation and thrombosis.”

The major capsid protein in ChAdOx1 (vector only), hexon, is considerably negatively charged (119). Brownian dynamics (BD) simulations of PF4 with the ChAdOx1 capsid structure revealed that PF4 binds to ChAdOx1 in the inter-hexon space (119), suggesting that the ChAdOx1 adenoviral vector used by Oxford/AstraZeneca may bind PF4 directly. This binding is inhibited by the addition of heparin. Additionally, the BD simulations showed that PF4 binds to ChAdOx1 more frequently than to Ad26. This may reflect real-world data where the incidence of VITT after ChAdOx1 nCoV-19 vaccination is much greater compared to post-vaccination with Ad26.COV2.S (Table 1). However, the authors warn that BD simulations do not account for flexibility of protein interactions and therefore it is not reliable to determine whether stable complexes are formed (119). Greinacher et al. also showed that ChAdOx1 nCoV-19 hexon, PF4, and VITT patient-derived anti-PF4 IgG form complexes on platelet surfaces, and that charge potential of AV hexon/PF4 complexes is neutralized by the addition of heparin, mirroring the simulation data previously mentioned (68).

ChAdOx1 nCoV-19 or Ad26.COV2.S administration almost exclusively accounts for the world’s cases of confirmed VITT. However, it has still not been resolved why vaccination with ChAdOx1 nCoV-19 disproportionately leads to most of the cases of VITT. Since Nicolai et al. showed that thrombocytopenia caused by adenoviral vectors is subtype-dependent (117), it is prudent to perform more *in vivo* studies directly comparing ChAdOx1 nCoV-19 and Ad26.COV2.S or their corresponding vectors, human adenovirus 26 and chimpanzee adenovirus Y25, respectively.

#### 5.2.3. Contamination with host-cell and/or adenoviral proteins act as a potential polyanion

ChAdOx1 nCoV-19 and Ad26.COV2.S are propagated in HEK293 and PER.C6, respectively. Comparative analysis of ChAdOx1 nCoV-19 and Ad26.COV2.S showed that ChAdOx1 nCoV-19 contains significantly more impurities, including HEK293 and adenoviral proteins (56, 57). Five lots of ChAdOx1 nCoV-19 tested showed contamination with proteasome 20S subunit beta 5, and showed increased proteasomal activity compared to Ad26.COV2.S and control. Platelets are immune sensors as well as hemostatic mediators, and may be sensitive to contact with cellular debris potentially introduced by vaccination with ChAdOx1 nCoV-19 and to a much lesser degree, Ad26.COV2.S (56). It is possible that impurities such as AV hexons and HEK293 debris can generate an inflammatory environment, activate platelets, and thus raise PF4 plasma levels. This idea is substantiated by ChAdOx1 nCoV-19 vaccination causing more inflammation and platelet activation than mRNA-based vaccines (59–61), which are free of animal or viral-derived contamination (59–61). It is also possible that antigens found in the HEK293 impurities complex with PF4, leading to the generation of PF4/polyanion neoantigens and precipitating an anti-PF4 response. However, this hypothesis has yet to be further investigated. In addition, this hypothesis cannot explain why VITT is observed in people vaccinated with mRNA-based COVID-19 vaccines (27–30), as mRNA-based vaccines are free of the aforementioned impurities. Since incidence of VITT after mRNA-based vaccination is at least two magnitudes lower compared to AV-based vaccination, it has been posited that reactions to the mRNA-based COVID-19 vaccines which resemble VITT may be reflecting the background rate of spontaneous HIT, where heparin is not required to mount an anti-PF4 response (10). Nevertheless, it is possible that contamination in ChAdOx1 nCoV-19 serves as an adjuvant to potentiate the anti-PF4 response and is therefore not required for the initiation of PF4/polyanion complex formation, which could explain the greater incidence observed in ChAdOx1 nCoV-19 versus Ad26.COV2.S vaccination.

#### 5.2.4. Anti-PF4 IgG in VITT may share a common polymorphism

In five confirmed VITT patients, Wang et al. found an IgG light chain stereotypy, wherein the variable light chain region was encoded by the identical IGLV3-21*02 gene subfamily for all five VITT patients (120). Notably, this dominant stereotyped expression of IGLV3-21*02 may be considered a “unique fingerprint” of anti-PF4 IgGs in VITT, as it has not been observed in any other serum antibody responses to date (120). For the first time, screening for a genetic risk factor for VITT, specially the IGLV3-21*02 light chain, may be meaningful. However, a larger cohort of VITT patients must be examined to confirm the viability of these findings.

Wang et al. mention that IGLV3-21*02 has differentially high expression in European populations (120). Specifically, the frequency of IGLV3-21*02 is high in those with western and northern European ancestry, including in Scandinavians (121). Interestingly, the highest rates of VITT are reported in Scandinavian countries, such as Denmark or Norway (42, 64), which warrants further investigation into the correlation between IGLV3-21*02 and susceptibility to VITT.

## 6. The mechanism of thrombosis and thrombocytopenia in the VITT milieu

The anti-PF4 response may have evolved as an ancient host defense mechanism (122): for example, PF4 can bind to negatively-charged cell walls of various bacterial species, in which bacteria are tagged by PF4 and opsonized by anti-PF4 antibodies, which significantly enhances their phagocytosis (122). The concept of this mechanism applies to VITT or anti-PF4 disorders. VITT, however, features an aberrant anti-PF4 response, where PF4 complexes with some exogenous polyanion, thus mounting an immune response against it, and thereby forming PF4/polyanion immune complexes as well as anti-PF4 IgG which drive VITT pathology.

### 6.1. VITT pathology is FcγRIIa-mediated

**Figure 2:**
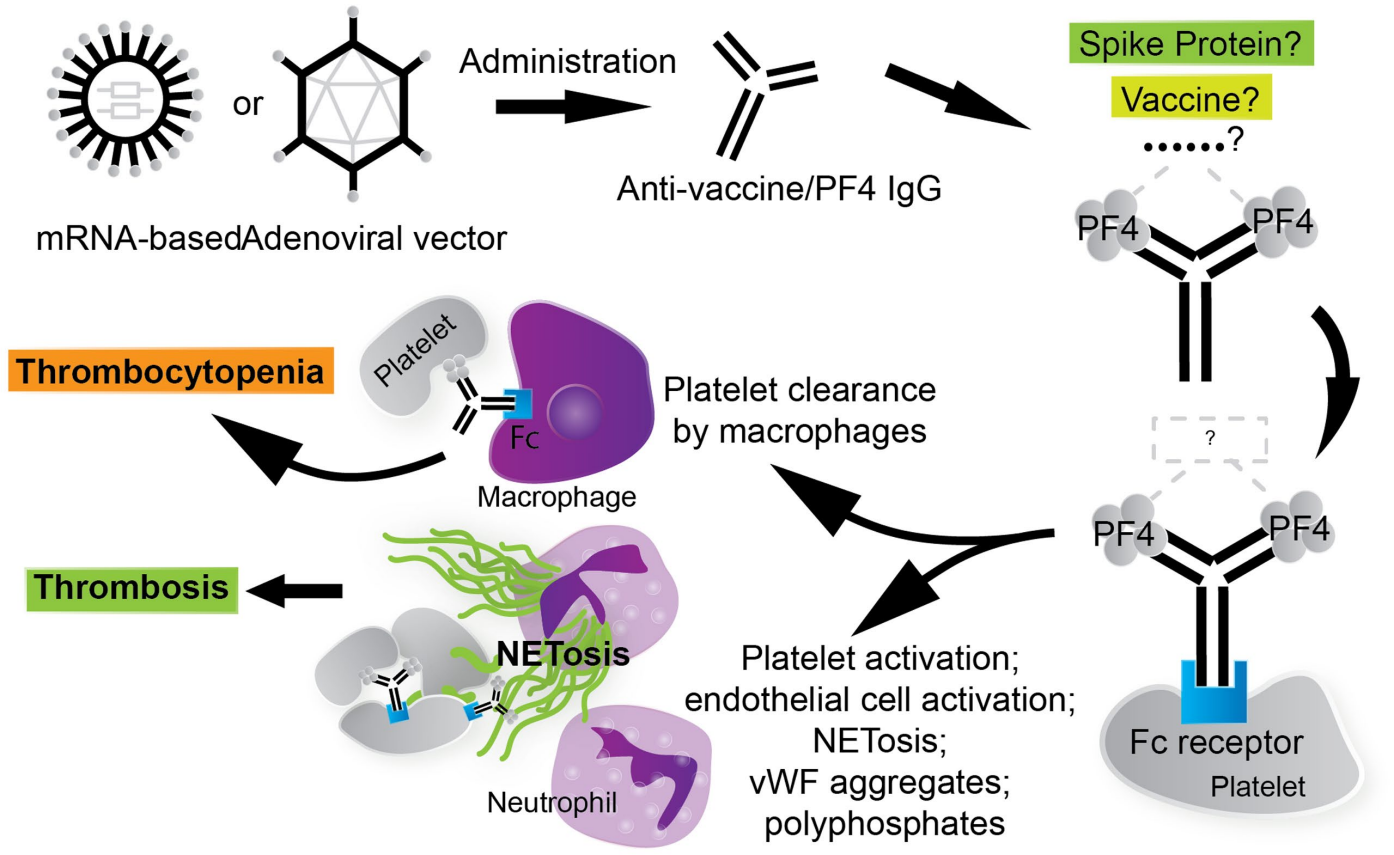
The thrombotic, thrombocytopenic cascade in VITT is anti-PF4 driven. COVID-19 vaccines, especially AV vaccines, can trigger the formation of anti-PF4/polyanion antibodies. The stimulus for the formation of VITT anti-PF4 antibodies is currently under investigation. The thrombotic and thrombocytopenic sequelae that follow are Fc fragment of IgG, low affinity IIA, receptor (FcγRIIa)-mediated. Leukocytes participate in the VITT milieu considerably, where neutrophils contribute to thrombosis via the generation of neutrophil extracellular traps (NETs) and circulating phagocytes, such as macrophages, clear platelets via immune-complex mediated clearance. Thrombosis in VITT features classic thrombotic hallmarks as well, such as von Willebrand factor (vWF) aggregates, which contribute to NETosis and are stabilized by PF4/polyanion complexes.

The thrombotic and thrombocytopenic sequelae in VITT are understood to be mediated by Fc fragment of IgG, low affinity IIA, receptor (FcγRIIa), also known as CD32a. Monoclonal antibody IV.3, which targets FcγRII, significantly blocks PF4-dependent platelet activation (25), suggesting that the platelet-activating anti-PF4 antibodies in the VITT milieu function through FcγRIIa. FcγRIIa is widely expressed on many type of cells, including platelets, neutrophils, and monocytes (123). FcγRIIa mediates many cellular defense functions including phagocytosis, pro-inflammatory cytokine release, and clearance of immune complexes via activation of the immunoreceptor tyrosine-based activation motif (ITAM) (123, 124). FcγRIIa can potently activate platelets when bound by the Fc of anti-platelet antibodies (125) and has been implicated in platelet depletion via circulating phagocytes through immune-complex mediated clearance (126, 127). Hence, a thrombotic thrombocytopenia can manifest in an FcγRIIa-dependent manner (Figure 2). Anti-PF4 antibodies crosslink FcγRIIa receptors on platelets, leading to platelet activation, aggregation, and degranulation (128).

### 6.2. Neutrophil extracellular traps mediate platelet activation and thrombosis

FcγRIIa is also expressed on circulating neutrophils and monocytes, which may explain the generation of neutrophil extracellular traps (NETs) and hyper-inflammatory environment in VITT patients, thus further promoting a hypercoagulable state (75, 128, 129). NETs are clusters of extracellular fibers composed primarily of DNA, granular proteins, and histones which are released by neutrophils in order to aid in the capture and killing of exogenous pathogens, while minimizing host cell damage (130). NETs can promote thrombosis because they act as a structural scaffold for the adhesion of platelets and platelet adhesion ligands such as vWF and fibrinogen (131). In addition to containing platelet adhesion ligands, NETs also harbor platelet activating agonists such as histone H3 (132). Studies have shown that cerebral thrombi in VITT are neutrophil-rich (68, 129, 133). The induction of NETosis by platelets pre-treated with anti-PF4 antibodies depends on neutrophil–platelet interactions via neutrophilic P-selectin glycoprotein ligand-1 (PSGL-1 or CD162) and P-selectin (CD62p) (134, 135). Inhibition of CD62p or PSGL-1 attenuates NETosis formation and DNA release by neutrophils (134, 135). Neutrophil activation and subsequent NETosis are vital for development of thrombosis in HIT (134, 136). While platelet activation is not essential in PF4/polyanion complex-induced NETosis in HIT (134), platelets are required to induce NETosis in VITT (68). The addition of PF4 to VITT patient serum or to affinity-purified anti-PF4 IgG VITT antibodies induces NETosis under the presence of neutrophils and platelets, strictly (68). This suggests that the interaction of platelets and neutrophils is essential to thrombus formation in the context of NETosis in VITT.

Using a double transgenic (FcγRIIa+/hPF4+) mouse model of VITT and a flow microfluidics system, Leung et al. showed that VITT antibodies directly stimulate neutrophils to release NETs and thereby induce thrombus formations containing platelets, neutrophils, fibrin, extracellular DNA, and citrullinated histone H3 (132). Both thrombocytopenia and thrombosis were prevented with FcγRIIa inhibition via IV.3, but only thrombosis and not thrombocytopenia was abrogated by NETosis inhibitor GSK484 or the genetic knockout of peptidylarginine deiminase 4 (PAD4) in the FcγRIIa+/hPF4+ mice (132). PAD4 citrullinates histones and is required for chromatin decondensation and expulsion, and therefore NETosis (137). This suggests that thrombosis may be indirectly distinct from thrombocytopenia in VITT (132), and that thrombosis and not both thrombosis and thrombocytopenia may be targeted specifically.

VITT characteristically features intracranial thrombotic involvement, but the mechanism of the pervasive development of CVST in VITT is unknown. It is possible that NETosis, which drives thrombosis in VITT, is responsible. Preliminary findings found that endothelium in the central nervous system lowly expresses deoxyribonuclease I (DNase I) (39). Since NETs are DNA-rich and DNA promotes thrombosis formation in NETs, impaired DNase I-mediated degradation of NETs may be a key contributor to CVST in VITT (138).

### 6.3. Other potential mechanisms mediating platelet activation and thrombosis

vWF has been proposed to be a pathogenic contributor to platelet-rich thrombi in VITT (139). Notably, vWF can bind to immobilized extracellular DNA released from neutrophils (e.g., NETs) and mediate neutrophil adhesion to endothelium under physiological blood flow (140). PF4/polyanion complexes can directly activate endothelial cells, which enhances the release of vWF and the expression of adhesion molecules such as E-selectins and P-selectins (107). PF4 can bind vWF directly and the subsequently-formed PF4/vWF complex is recognized by anti-PF4 antibodies, leading to the generation of PF4/vWF immune complexes (141). Platelets can then be activated by the PF4/vWF immune complexes in an FcγRIIA and GPIb-IX dependent manner (141). PF4 can bind to polymeric strings of vWF, as opposed to monomeric vWF. These complexes are not uniformly distributed along vWF strings, but aggregate along thicker vWF strands (142). PF4 has been found to inhibit proteolytic activity of ADAMTS13 (143), an endogenous enzyme that digests vWF multimers. Furthermore, PF4/vWF immune complexes seem to stabilize vWF multimers, which shields vWF strings from proteolysis by ADAMTS13 and promotes thrombosis (139, 141). PF4, therefore, facilitates the stabilization of vWF strings and binds to them, effectively establishing a positive feedback relationship between the release of PF4 and the stabilization of vWF strings in the thrombotic milieu.

## 7. Measuring platelet-activating anti-PF4 IgG is critical for diagnosing VITT

Precisely measuring platelet-activating anti-PF4 antibody titers is critical for efficiently diagnosing VITT. However, current diagnostic methods vary in their sensitivity or specificity in detecting pathologically relevant anti-PF4 titers. As of Q4 2022, there is no gold standard diagnostic test for VITT and according to the largest scale multicenter study for VITT anti-PF4 detection to date; current anti-PF4 enzyme-linked immunosorbent assays (ELISAs) produce false positive and false negative results (78). Although it is more time-intensive and uses more resources, multiple ELISAs from different manufacturers can be used and if multiple high optical density (OD) values are derived (e.g., OD > 2.0), then the risk of a false positive diagnosis is lower, but further confirmatory functional testing is still advised (78). Since HIT patients have polyclonal anti-PF4 populations while VITT patients present with oligoclonal or monoclonal anti-PF4 populations (65), it is possible that the ELISAs made for HIT anti-PF4 detection that are used to detect novel anti-PF4 antibodies are either too sensitive or may not even detect the novel anti-PF4 in VITT. The majority of methods used to diagnose VITT include ELISAs, rapid tests such as chemiluminescence immunoassays (CLIAs), enzyme immunoassays (EIAs), PF4-^14^C-serotonin release assay (PF4-^14^C-SRA), and washed platelet functional assays such as the PF4-induced platelet activation (PIPA) and PF4-induced flow cytometry-based platelet activation (PIFPA) assays. Diagnostic methods for VITT, particularly the immunoassays, are generally HIT-based, and have pros and cons, which are summarized in Table 2.

**Table 2:** Pros and cons of diagnostic assays used for VITT.

Assay	Sensitivity	Specificity	Pros	Cons
IgG ELISAs	70–94% (144)	77–100% (144)	OD > 2.0 likely reflects a true positive; relatively quick turnaround time	OD < 2.0 is ambiguous; multiple ELISAs preferred; further functional testing should follow a positive result
HemosIL AcuStar HIT-IgG CLIA	5.9% (144)	100% (144)	Rapid turnaround time (30–60 min)	Low sensitivity for VITT; should be avoided due to risk of false negatives
EIAs	100% (145)	95–97% (145)	Can provide a “likely” VITT diagnosis (146)	Further functional testing should follow a positive result
PF4-^14^C-SRA	46% (145)	100% (145)	Improves on the standard SRA, which is prone to producing false negatives for VITT (147)	Time-consuming, utilizes radiation, expensive (148); can still produce false negatives for VITT
PIPA	100% (146)	100% (146)	Functionally (pathologically) relevant	Multi-step; requires specific laboratory equipment
PIFPA	100% (146)	100% (146)	Functionally (pathologically) relevant	Multi-step; requires specific laboratory equipment

Handtke et al. recommend a diagnostic algorithm in which a positive anti-PF4/heparin (polyanion) EIA warrants a likely VITT diagnosis, which should be confirmed with a PIPA or PIFPA (146). A negative anti-PF4/polyanion EIA does not reject a VITT diagnosis but deems it unlikely. Like ELISAs, greater anti-PF4/heparin IgG EIA OD readings predict higher probability of platelet-activating antibodies in VITT patient sera (149). Still, using EIA, 6.7% of participants tested positive for anti-PF4 antibodies in a study of 281 participants inoculated with ChAdOx1 nCoV-19 or BNT162b2, albeit with low OD values (77). None of the individuals in this study exhibited symptoms of VITT. Most interestingly, when diluting VITT patient sera at 1/4 to 1/10 dilution, EIA OD values increase, unlike in HIT. This suggests that an optimal complex formation depends on the stoichiometric ratio of PF4 and anti-PF4 antibodies in VITT (149).

Kanack et al. have reported an un-complexed PF4 ELISA which sensitively and specifically detects anti-PF4 VITT antibodies and can differentiate spontaneous HIT from VITT (150). Additionally, they found that the quantification of thrombospondin-1 (TSP1) released from PF4/heparin-treated cryopreserved platelets is accurate for detecting and potentially differentiating VITT and HIT patient antibodies (151). Since this assay is practical to conduct in most hospitals due to shelf-life and accessibility of cryopreserved platelets, it has the potential to change the standard of diagnosing VITT.

## 8. Current therapies for VITT

Rapid therapeutic intervention is critical in saving the lives of VITT patients. Disturbingly, although anti-PF4 antibodies are transient in most VITT patients (152), persistent anti-PF4 titers and/or elevated D-dimer have been reported in some VITT patients, spanning over 12 weeks from initial diagnosis (152, 153), which highlights the need for specific VITT treatment for both acute and chronic settings. As discussed above, VITT is FcγRIIa-mediated. Therefore, immune-suppressive therapy is one effective strategy. The American Society of Hematology as well as other expert groups (34, 154–156) have suggested the first line treatment for confirmed VITT should be IV immunoglobulin (IVIG) at a dose of 1 g/kg daily for two days. Corticosteroids, like dexamethasone, can be used as supplements to IVIG (15, 155). In comparison to the non-use of IVIG, the use of IVIG significantly reduced mortality in two separate studies of 70 and 99 VITT patients (157, 158), accordingly validating the efficacy of this first line treatment.

Heparin has been used in VITT patients with positive outcomes but its use in VITT is still debated (34, 65, 67, 153, 159, 160). Though heparin has been found to inhibit platelet activation in PF4-stimulated VITT sera (25, 65, 67, 151) and dissociate PF4/polyanion complexes in VITT (76, 159), which is possibly due to its competition with VITT anti-PF4 antibodies to bind the neoepitope of PF4 (76). Hesitance to use heparin in the treatment of VITT patients is possibly due to the association of VITT with HIT, where heparin is contraindicated. Scutelnic et al. and the meta-analysis by Kim et al. found no significant difference in mortality between groups of VITT patients receiving heparin-or non-heparin-based anticoagulation treatments (37, 158). Regardless, the risk of heparin triggering HIT in the VITT milieu is a possibility and remains unexplored.

The clinical studies comparing heparin to non-heparin anticoagulation use in VITT patients have or may have multiple confounders, including the dual use of heparin and non-heparin anticoagulation in the same patient (37, 157, 158), concurrent use of IVIG (37, 157, 158), and/or differences in patient acuity. Nevertheless, in comparison to VITT patients who did not receive any anticoagulants or heparin, Perry et al. reported that patients who received non-heparin anticoagulation had reduced mortality (157). Accordingly, non-heparin anticoagulation such as thrombin inhibitors (e.g., argatroban) and factor Xa inhibitors (e.g., fondaparinux) have been recommended and have been used to mitigate thrombotic events in VITT (25, 34, 154–156, 161). The indirect thrombin inhibitor, danaparoid, has also been used with positive outcomes (162). Interestingly, danaparoid and heparin, but not fondaparinux or argatroban, interfere with anti-PF4 binding to PF4 *in vitro* (163). A non-specific but potentially robust treatment in VITT may be to target fibrinolysis, since enhancing fibrinolysis was shown to be efficacious in a mouse model of HIT, even compared to IV.3 usage (164). Additionally, eculizumab, a complement component 5 (C5)-cleavage inhibitor, has been used successfully when recurrent thromboembolic events were still observed after IVIG (25, 165). The inhibition of C5 cleavage may be efficacious in VITT because complement components, particularly C5 and its cleavage product, C5a, are implicated in NET-or histone-induced thrombosis (166, 167).

Interestingly, preventing tetramerization of PF4 attenuates anti-PF4 IgG-induced platelet activation and platelet aggregation *in vitro* and thrombus progression *in vivo* in HIT (86). Although preventing PF4 tetramerization is unexplored in the context of VITT at this time, small molecule inhibitors have been proposed for HIT, which effectively inactivate PF4 to form immune complexes and even render preformed PF4-containing immune complexes unable to activate FcγRIIa (168).

Our lab recently found that thymidine phosphorylase (TYMP) plays an important role in platelet activation and thrombosis (169–171). Studies have also found that TYMP is significantly increased in the plasma (172, 173), lungs (174), platelets (175), and monocytes (176) of patients with COVID-19, especially in the early phase. TYMP binds to and reduces activity of Src family kinases (SFKs), such as Lyn (169, 177). This inhibits the transfer of a phosphate group from Lyn (or from other SFKs, such as Fyn and Yes) to the immunoreceptor tyrosine-based inhibitory motif (ITIM) of platelet endothelial cell adhesion molecule 1 (PECAM-1; CD31) and attenuates PECAM-1 activation in platelets (169). Activation of PECAM-1 inhibits platelet glycoprotein VI (GPVI) and GPIb/V/IX signal transductions and thus inhibits thrombosis, which is mediated by reducing the rapid tyrosine phosphorylation of the ITAM of the FcR gamma chain that is associated with GPVI or GPIb (178, 179). Since FcγRII also signals through the activation of the ITAM (123, 124), which TYMP indirectly can regulate, targeting TYMP may also be an effective treatment for VITT. To the interest of this hypothesis, a selective inhibitor of TYMP, tipiracil, has been approved by the FDA for another clinical indication, and tipiracil has been reported to reduce SARS-CoV-2 SP production (180) as well as inhibit thrombosis in mice (171).

## 9. Summary

In summary, it is possible that the true mechanism of VITT induction is an amalgam of current hypotheses, similar to the three-hit mechanism that De Michele et al. suggest (110). The AV COVID-19 vaccines are potentially facilitating an ideal environment for the formation of PF4/polyanion immune complexes: (1) PF4 may be increased in the blood via interaction of platelets with AV hexon and/or SARS-CoV-2 SP causing platelet degranulation; (2) a PF4/polyanion complex consisting of PF4, SP, and GAGs can be potentially formed by a soluble form of aberrantly spliced ChAdOx1 nCoV-19 SP and/or S1 subunits in the bloodstream or SP expressed via the endothelium post-AV COVID-19 vaccination (Figure 1); or AV hexons or proteins found in AV vaccine impurities via host cell and/or viral contamination may form complexes with PF4 as well; (3) once PF4/polyanion complexes are formed, conformational changes in PF4 tetramers occur, leading to the exposure of a neoepitope and thus, anti-PF4 antibodies or PF4/polyanion immune complexes can be formed which cause thrombotic and thrombocytopenic sequelae (Figure 2).

## 10. Conclusion

The experimental data and hypotheses discussed and analyzed in this review help paint a mechanistic picture that may elucidate the pathogenesis of VITT, which will not only improve its diagnosis and treatment, but will establish a precedent for rigorous vaccine design as well.

## Author contributions

RR and WL wrote and edited the manuscript. AG-S provided comments and criticized the manuscript. All authors contributed to the article and approved the submitted version.

## Funding

This work is supported by the Marshall University Institute Fund (to WL), the National Institutes of Health R15HL145573 (PI: WL), the American Heart Association Predoctoral Fellowship (23PRE1018686 to RR), the West Virginia IDeA Network of Biomedical Research Excellence WV-INBRE (P20GM103434), and the West Virginia Clinical and Translational Science Institute-Pop-Up COVID-19 Fund (to WL) supported by the National Institute of General Medical Sciences (U54GM104942). The content is solely the responsibility of the authors and does not necessarily represent the official views of the National Institutes of Health.

## Conflict of interest

The authors declare that the research was conducted in the absence of any commercial or financial relationships that could be construed as a potential conflict of interest.

## Publisher’s note

All claims expressed in this article are solely those of the authors and do not necessarily represent those of their affiliated organizations, or those of the publisher, the editors and the reviewers. Any product that may be evaluated in this article, or claim that may be made by its manufacturer, is not guaranteed or endorsed by the publisher.
